# A multifactorial approach including tumoural epidermal growth factor receptor, p53, thymidylate synthase and dihydropyrimidine dehydrogenase to predict treatment outcome in head and neck cancer patients receiving 5-fluorouracil

**DOI:** 10.1038/sj.bjc.6690297

**Published:** 1999-04

**Authors:** M C Etienne, X Pivot, J L Formento, R J Bensadoun, P Formento, O Dassonville, M Francoual, G Poissonnet, X Fontana, M Schneider, F Demard, G Milano

**Affiliations:** Centre Antoine Lacassagne, 33 Av de Valombrose, Nice, Cedex 2, 06189, France

**Keywords:** head and neck cancer, induction treatment, epidermal growth factor receptor, thymidylate synthase, p53, dihydropyrimidine dehydrogenase

## Abstract

The prognostic value of tumoural epidermal growth factor receptor (EGFR), p53, thymidylate synthase (TS) and dihydropyrimidine dehydrogenase (DPD) was analysed on 82 advanced head and neck cancer patients (71 men, 11 women; mean age 59). Induction treatment was cisplatin–5-FU ± folinic acid (61 patients, Chem group) or concomitant cisplatin–5-FU–radiotherapy (21 patients, RChem group). EGFR (binding assay), p53 protein (Sangtec immunoluminometric assay), TS and DPD activities (radioenzymatic assays) were measured on biopsies obtained at time of diagnosis. Significant positive correlation was demonstrated between p53 and EGFR. In the RChem group, p53 was higher in non-complete responders (median 1.03 ng mg^−1^) than in complete responders (median 0.08 ng mg^−1^) (*P* = 0.057). Univariate Cox analyses stratified on treatment group showed that specific survival (33 events) was significantly related to T staging, p53 taken as continuous or categorial (below vs over 0.80 ng mg^−1^) variable, and EGFR (below vs over 220 fmol mg^−1^); survival increased when EGFR and p53 were below thresholds. Multivariate stepwise analysis including T staging, EGFR and p53 revealed that T staging and EGFR were independent predictors of survival; relative risks were 3.68 for T staging and 2.65 for EGFR. Overall, EGFR remained an independent prognostic factor when response to treatment and T staging were considered in the multivariate analysis. © 1999 Cancer Research Campaign


*British Journal of Cancer* (1999) **79**(11/12), 1864–1869
© 1999 Cancer Research Campaign
Article no. bjoc.1998.0297

### A multifactorial approach including tumoural epidermal growth factor receptor, p53, thymidylate synthase and dihydropyrimidine dehydrogenase to predict treatment outcome in head and neck cancer patients receiving 5-fluorouracil

**MC Etienne, X Pivot, JL Formento, RJ Bensadoun, P Formento, O Dassonville, M Francoual, G Poissonnet, X Fontana, M Schneider, F Demard and G Milano**

Centre Antoine Lacassagne, 33 Av de Valombrose, 06189 Nice Cedex 2, France

**Summary** The prognostic value of tumoural epidermal growth factor receptor (EGFR), p53, thymidylate synthase (TS) and dihydropyrimidine dehydrogenase (DPD) was analysed on 82 advanced head and neck cancer patients (71 men, 11 women; mean age 59). Induction treatment was cisplatin–5-FU ± folinic acid (61 patients, Chem group) or concomitant cisplatin–5-FU–radiotherapy (21 patients, RChem group). EGFR (binding assay), p53 protein (Sangtec immunoluminometric assay), TS and DPD activities (radioenzymatic assays) were measured on biopsies obtained at time of diagnosis. Significant positive correlation was demonstrated between p53 and EGFR. In the RChem group, p53 was higher in non-complete responders (median 1.03 ng $mg^{-1}$) than in complete responders (median 0.08 ng $mg^{-1}$) ($P = 0.057$). Univariate Cox analyses stratified on treatment group showed that specific survival (33 events) was significantly related to T staging, p53 taken as continuous or categorial (below vs over 0.80 ng $mg^{-1}$) variable, and EGFR (below vs over 220 fmol $mg^{-1}$); survival increased when EGFR and p53 were below thresholds. Multivariate stepwise analysis including T staging, EGFR and p53 revealed that T staging and EGFR were independent predictors of survival; relative risks were 3.68 for T staging and 2.65 for EGFR. Overall, EGFR remained an independent prognostic factor when response to treatment and T staging were considered in the multivariate analysis.

**Keywords:** head and neck cancer; induction treatment; epidermal growth factor receptor; thymidylate synthase; p53; dihydropyrimidine dehydrogenase

For patients with head and neck squamous cell carcinoma (HNSCC), overall survival remains low. Identification of reliable tumour markers that reflect tumour aggressiveness could thus provide useful tools for choosing more or less aggressive treatment. In this cancer localization, induction treatment provides marked tumour responses and numerous data have shown that overall survival was significantly improved in patients with complete response (Spaulding et al, 1994). This link strengthens the need to identify those patients who are likely to exhibit a more favourable evolution following induction treatment. Investigations at the tumoural level can provide relevant information regarding not only intrinsic biological aggressiveness, but also its potential responsiveness to loco-regional curative treatments.

Previous studies by Maurizi et al (1996) and us (Dassonville et al, 1993) have shown that tumoural expression of epidermal growth factor receptor (EGFR) carries a strong prognostic value in head and neck cancer. Mutations of the tumour suppressor gene *p53* have been implicated in the pathogenesis of HNSCC (Greenblatt et al, 1994). As recently pointed out by Raybaud-Diogène and colleagues (1996), the clinical significance of *p53* gene alterations in HNSCC is currently being debated with some studies showing a link between *p53* mutations and poor clinical outcome whereas other investigators did not find such a relationship. *p53* plays a dual role by controlling both the tumoural cell dynamics at the G1/S checkpoint and the programmed cell death (Harris, 1996). Interestingly, a recent study suggested that mutations of the *p53* gene are associated with an increased risk of loco-regional failure in patients with advanced HNSCC treated with radiotherapy (Koch et al, 1996). Most induction chemotherapy protocols in HNSCC include 5-fluorouracil (5-FU) (Vokes et al, 1993). Previous studies by our group have shown that tumoural activity of dihydropyrimidine dehydrogenase (DPD), the first key enzyme of 5-FU catabolism, can be a determining factor for predicting 5-FU responsiveness in HNSCC (Etienne et al, 1995). Also, Johnston and co-workers (1997) concluded from a retrospective study that characterization of tumoural thymidylate synthase (TS) expression may be of value in identifying HNSCC patients who would benefit from 5-FU-based induction chemotherapy.

In total, the above-cited parameters are potential predictors of 5-FU treatment outcome. So far, no study has tested their prognostic values using a multifactorial approach. Our purpose was thus to conduct a multifactorial study including the above potential tumoural indicators of biological aggressiveness and treatment

*Received 15 May 1998*
*Revised 24 August 1998*
*Accepted 24 August 1998*

*Correspondence to*: G Milano responsiveness to predict outcome in advanced HNSCC patients receiving 5-FU-based induction chemotherapy. EGFR, *p53*, TS activity and DPD activity were determined on tumoural biopsies obtained at time of diagnosis in 82 patients.

### MATERIAL AND METHODS

### Patients

This study was conducted on 82 advanced head and neck cancer patients (71 men, 11 women; mean age 59 ranging from 40 to 76). Tumour localizations were as follows: oropharynx (42), hypopharynx (20), oral cavity (11), larynx (7) and multiple localizations (2). Description of T staging and N staging (UICC/TNM criteria) is presented in Table 1.

Initial treatment was either induction chemotherapy (Chem) with cisplatin–5-FU ± folinic acid (61 patients), or concomitant radio-chemotherapy (RChem, 21 patients). Induction chemotherapy consisted of 3 cycles administered at 3-week intervals. Cisplatin (100 mg $m^{-2}$, 1 mg $min^{-1}$ intravenously) was administered on day 1, with hyperhydration given for 2 h before and 4 h after cisplatin administration. 5-FU ± folinic acid was given as a 5-day continuous infusion from day 2 to day 6, with individual 5-FU dose adaptation on day 4, if necessary, based on pharmacokinetics (Santini et al, 1989). Initial 5-FU dose was 1000 mg $m^{-2}$ $day^{-1}$ when 5-FU was administered without folinic acid (42 patients) and 500 mg $m^{-2}$ $day^{-1}$ when 5-FU was administered in combination with folinic acid (200 mg $m^{-2}$ $day^{-1}$ of the pure *l* form, 19 patients). Concomitant RChem consisted of three cisplatin–5-FU courses at 3-week intervals, associated with continuous radiotherapy given as two daily fractions of 1.2 Gy on primary tumour and satellite nodes (5 days per week for 7 weeks) using Cobalt 60 or linear accelerator photons (total dose of radiotherapy 80.4 Gy). In the RChem group, cisplatin and 5-FU were given as described above, with a 5-FU dose of 750 mg $m^{-2}$ $day^{-1}$. Following induction treatment, 64 patients received a loco-regional treatment consisting of radiotherapy alone (33 patients), surgery plus radiotherapy (22 patients) or other modalities (nine patients).

Clinical response (tumoural and global) was evaluated 2 weeks after completion of initial treatment, by calculating the product of the two perpendicular lesion diameters measured by endoscopy (UICC criteria). Complete response (CR) corresponded to the disappearance of all clinically visible or palpable lesions. Partial response (PR) was defined as tumour regression greater than 50%. Treatment failure corresponded to tumour regression of less than 50% or tumour progression.

Duration of survival was calculated from the date of initial diagnosis. For specific survival, the end-point was any cancer-related death. Only three patients were lost to follow-up and were considered as censored observations in survival analyses. At time of analysis, 46 patients had died. Median follow-up was 25 months for the whole population and 40 months for alive patients.

**Table 1** Description of T staging and N staging (UICC criteria)

| | N0 | N1 | N2 | N3 | Total |
|---|---|---|---|---|---|
| T1 | 0 | 0 | 1 | 1 | 2 |
| T2 | 2 | 1 | 1 | 2 | 6 |
| T3 | 14 | 5 | 16 | 3 | 38 |
| T4 | 3 | 6 | 21 | 5 | 35 |
| Total | 19 | 12 | 39 | 11 | 81[2] |

[2]TN staging not available for one patient.

### Tumoural investigations

Tumoural biopsies correspond to a part of the tumour sample obtained for diagnosis purpose (30–70 mg). Tumour samples were obtained at time of diagnosis and were stored in liquid nitrogen until assayed. Tumoural biopsies were homogenized in Tris-HCl buffer. Differential centrigurations allowed the separation of crude membranes from the cytosol. DPD activity, TS activity and p53 protein content were measured on the cytosol. EGFR was assayed on the membrane preparation of the same tumoural sample using a method that we previously published (Santini et al, 1991), based on competition between $^{125}$I-EGF and unlabelled EGF (human recombinant EGF); sensitivity limit was 1 fmol $mg^{-1}$ protein and inter-day reproducibility was 11%. DPD activity was measured by means of a radioenzymatic assay initially described by Harris et al (1990) and modified by us (Beck et al, 1994), using $^{14}$C-5-FU as substrate; sensitivity limit was 10 pmol $min^{-1}$ mg-1 protein and inter-day reproducibility was 10%. TS activity was assayed by means of the tritium-release assay (Spears et al, 1988), modified by us, with $^{3}$H-dUMP as substrate; sensitivity limit was 10 fmol $min^{-1}$ $mg^{-1}$ protein and inter-day reproducibility was 15%. The p53 protein level (wild-type and mutated forms) was determined using a monoclonal two-site single incubation immunoluminometric assay (LIA-mat, Sangtec, Sweden), sensitivity limit was 0.002 ng $mg^{-1}$ and inter-day reproducibility was 8%.

### Statistics

Gaussian distribution was evaluated according to normal probability plot and Kolmogorov–Smirnov good-fit test. Comparison of tumoural parameters according to treatment response was performed using Mann–Whitney test in each treatment group separately. Overall survival and specific survival were computed using the Kaplan–Meier method. The influence of each predictor variable on specific survival was analysed according to the Cox proportional hazard regression stratified on treatment group (Chem vs RChem). Selection of predictors in multivariate analyses was done by stepwise analyses (forward and backward, based on the maximum partial likelihood estimates) stratified on treatment group. Probability for entry and removal was 0.05 and 0.10 respectively. T staging (T1-2-3 vs T4), N staging (N0 vs N1 vs N2 vs N3, with N0 being the reference group) and treatment response (PR + failure vs CR) were analysed as categorial variables. Continuous variables were EGFR (log 10), DPD (log 10), TS (log 10) and p53 (log 10). These variables were also analysed as categorial variables (0 if lower than threshold, 1 if greater) after estimation of a threshold defined as the significant one giving the greater Wald value in the Cox regression model. For that purpose, thresholds were tested within the first to third quartiles. For non-significant variables, the presently reported thresholds were the median values. Statistics were drawn up on SPSS software (Chicago, IL, USA).

### RESULTS

### Description of tumoural parameters

Wide variability was observed for all investigated tumoural parameters. EGFR (mean 356, median 110, extremes ND-3927

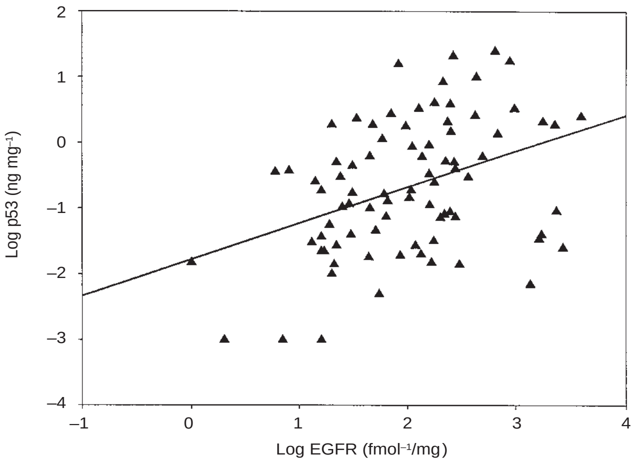

**Figure 1** Plot of the linear regression between log10 p53 and log10 EGFR ($n$ = 82, $r$ = 0.39, $P$ < 0.001)

**Table 3** Univariate Cox analyses for specific survival stratified on treatment group

| Covariable[a] | *P* | wald | $e^{\beta}$ |
|---|---|---|---|
| T staging (0 if T1-2-3, 1 if T4)[b] | 0.002 | 9.3 | 3.56 |
| log p53 | 0.033 | 4.6 | 1.45 |
| p53 (0 if below, 1 if over 0.80 ng $mg^{-1}$) | 0.017 | 5.8 | 2.37 |
| EGFR (0 if below, 1 if over 220 fmol $mg^{-1}$) | 0.024 | 5.1 | 2.33 |
| Global response (0 if PR + failure, 1 if CR)[b] | 0.0041 | 8.2 | 0.19 |

[a]TS and DPD analysed as continuous or categorial variables (below vs over median), EGFR (continuous variable) and N staging (N0 vs N1 versus N2 vs N3) were non-significant covariables. [b]With the exception of T staging (81 patients, 32 events) and global response (71 patients, 27 events), analyses were conducted on 82 patients (33 events). For any covariable, $e^{\beta}$ is equal to the risk of death of a patient presenting the value Xi divided by the risk of death of a patient presenting the value Xi-1, whatever the value of Xi. For categorial variables, $e^{\beta}$ represents the relative risk of death between the 2 classes of the variable. When $e^{\beta} > 1$, the risk of death rises when the variable increases; when $e^{\beta} < 1$, the risk of death decreases when the variable increases.

fmol $mg^{-1}$, $n$ = 82), p53 (mean 1.805, median 0.187, extremes ND-24.563 ng mg-1, $n$ = 82) and TS (mean 4320, median 1660, extremes ND- 81780 fmol $min^{-1}$ $mg^{-1}$, $n$ = 73) exhibited typical asymmetrical distribution, which fitted a Gaussian distribution after logarithm transformation. Tumoural DPD activity ranged from 27 to 567 pmol $min^{-1}$ $mg^{-1}$ (mean 168, median 147, $n$ = 72). A significant correlation was observed between log 10 EGFR and log 10 p53 (linear regression, $r$ = 0.39, $P$ < 0.001, $n$ = 82, Figure 1), the higher the EGFR level, the greater the p53 protein content. No relationship was seen between p53 protein content and TS activity, or between any of the other tested parameters.

### Analysis of response

In the Chem group (61 patients), tumoural response was the following: 25 CR, 22 PR, 14 failures; global response was assessable in 58 patients (15 CR, 23 PR, 20 failures). In the RChem group (21 patients), tumoural response was 14 CR, three PR and three failures (response non-assessable in one patient); global response was assessable in 13 patients only (eight CR, one PR, four failures). Table 2 describes EGFR, DPD, TS and p53 as a function of response in each treatment group. Globally, median TS was approximately twofold higher in patients with CR than in patients with PR or failure. However, this difference did not reach statistical significance. Regarding p53 protein, median values were higher in non-complete responding patients, although this observation was at the limit of significance in the RChem group only (1.033 ng $mg^{-1}$ in PR + failure patients vs 0.086 ng $mg^{-1}$ in CR patients, $P$ = 0.058).

### Analysis of survival

At time of analysis, 29 patients had died from their primary cancer, four from chemotherapy side-effects, two from another cancer, three from non-medical causes and eight from unknown causes. Taking into account all death causes, median overall survival was 36 months in the Chem group (30 events) and 12 months in the RChem group (16 events). Specific survival was analysed by considering the 33 head and neck cancer-related deaths (20 in the Chem group, 13 in the RChem group). Table 3 illustrates

Table 2 Description of tumoural parameters according to treatment response

| | | Chem group | | | RChem group | | |
|---|---|---|---|---|---|---|---|
| | | **CR** | | **PR + failure** | **CR** | | **PR + failure** |
| EGFR | N | 25 | | 36 | 14 | | 6 |
| ($fmol^{-1}/mg^{-1}$) | median | 104 | *P* = 0.81 | 77 | 229 | *P* = 0.97 | 213 |
| TS | N | 25 | | 31 | 11 | | 5 |
| ($fmol^{-1}/min^{-1}/mg^{-1}$) | median | 1810 | *P* = 0.29 | 1070 | 3270 | *P* = 0.13 | 1660 |
| DPD | N | 25 | | 30 | 11 | | 5 |
| ($pmol^{-1}/min^{-1}/mg^{-1}$) | median | 118 | *P* = 0.067 | 160 | 188 | *P* = 0.57 | 166 |
| p53 | N | 25 | | 36 | 14 | | 6 |
| ($ng^{-1}mg^{-1}$) | median | 0.116 | *P* = 0.29 | 0.282 | 0.086 | *P* = 0.058 | 1.033 |

P-value denotes the probability value of the Mann–Whitney test.

*© Cancer Research Campaign 1999*

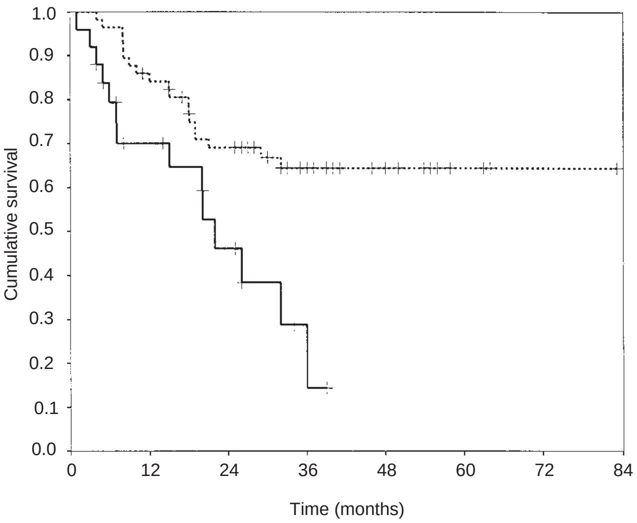

**Figure 2** Plot of cumulative specific survival according to tumoural p53 status. Solid line represents survival of patients with p53 above 0.80 ng $mg^{-1}$ (25 patients, 14 events). Dotted line represents survival of patients with p53 below 0.80 ng $mg^{-1}$ (57 patients, 19 events). Vertical bars indicate censored observations. *P*-value of the log-rank test was 0.0035

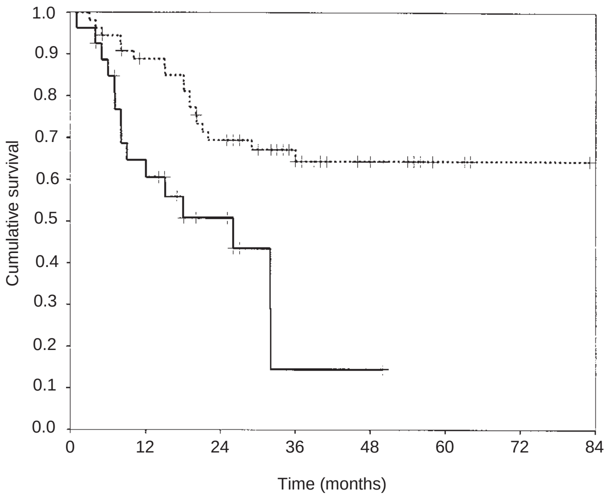

**Figure 3** Plot of cumulative specific survival according to EGFR status. Solid line represents survival of patients with EGFR above 220 fmol $mg^{-1}$ (27 patients, 15 events). Dotted line represents survival of patients with EGFR below 220 fmol $mg^{-1}$ (55 patients, 18 events). Vertical bars indicate censored observations. *P*-value of the log-rank test was 0.0012

univariate Cox analyses stratified on treatment group. The p53 protein content taken as a continuous variable was a significant predictor of specific survival, the lower the p53, the longer the survival ($P$ = 0.03, Table 3). The best discriminant p53 threshold was at 0.80 ng $mg^{-1}$ (Figure 2). EGFR was not significant as a continuous variable; however, survival was significantly increased in patients with EGFR below 220 fmol $mg^{-1}$ (Figure 3 and Table 3). Also, specific survival was significantly related to T staging and global response (Table 3). A multivariate stepwise analysis stratified on treatment group including T staging, EGFR and p53 status revealed that T staging and EGFR status were the only

**Table 4** Stepwise multivariate Cox analyses for specific survival stratified on treatment group

| Covariables | *P* | wald | $e^{\beta}$ |
|---|---|---|---|
| **A** T staging (0 if T1-2-3, 1 if T4) | 0.0014 | 10.2 | 3.68 |
| EGFR (0 if below, 1 if over 220 fmol $mg^{-1}$) | 0.0089 | 6.9 | 2.65 |
| p53 (0 if below, 1 if over 0.80 ng $mg^{-1}$) | NS | | |
| **B** T staging (0 if T1-2-3, 1 if T4) | 0.0027 | 9.0 | 3.72 |
| EGFR (0 if below, 1 if over 220 fmol $mg^{-1}$) | 0.0071 | 7.3 | 3.12 |
| p53 (0 if below, 1 if over 0.80 ng $mg^{-1}$) | NS | | |
| Global response (0 if PR + failure, 1 if CR) | 0.0051 | 7.8 | 0.15 |

Analysis **A** included tumoural parameters only (81 patients, 32 events). Analysis **B** included tumoural parameters and response to treatment (70 patients, 26 events). $e^{\beta}$ represents the relative risk of death between the 2 classes of the variable. When $e^{\beta} > 1$, the risk of death rises when the variable increases; when $e^{\beta} < 1$, the risk of death decreases when the variable increases.

independent predictors of survival (Table 4A). Relative risks were 3.68 (95% confidence interval 1.66–8.19) for T staging and 2.65 (95% confidence interval 1.28–5.50) for EGFR status. Finally, a stepwise analysis including response to treatment revealed that T staging, EGFR and response to treatment remained independent significant predictors of survival (Table 4B).

### DISCUSSION

During the last decade, induction treatment with chemotherapy or radiochemotherapy in locally advanced HNSCC has been shown to enhance loco-regional disease control, reduce metastases rate and, very importantly, preserve anatomic function (Dimery and Hong, 1993; Vokes et al, 1993; Lefebvre et al, 1996; Harari, 1997). It has been demonstrated that only those patients who achieve a complete response will benefit from the induction therapy in terms of survival (Spaulding, 1994). However, induction treatment strategies are not without severe side-effects and indicators of treatment efficacy and disease evolution would appear useful in orienting patients towards potentially successful treatment strategies. In line with the currently administered induction chemotherapy in advanced HNSCC, all patients of the present study received a cisplatin–5-FU-based induction treatment (Vokes, 1993). In the 61 patients with resectable disease, such induction chemotherapy was performed in a strategy of organ preservation. For the 21 patients exhibiting a strictly non-resectable tumour, a concomitant radiochemotherapy was administered. The objective of the present study was to analyse 5-FU sensitivity markers such as tumoral DPD and TS for which relevance for 5-FU sensitivity has previously been suggested (Etienne et al, 1995; Johnston et al, 1997) and to investigate disease aggressiveness indicators like EGFR (Dassonville et al, 1993) and p53 (Raybaud-Diogène et al, 1996). The drug efficacy markers presently investigated were centred on 5-FU responsiveness. One might wonder what is the role played by cisplatin in tumour response. Although some variability in cisplatin tumour sensitivity cannot be excluded, previous experimental (Etienne et al, 1991) and clinical studies (Dimery and Hong, 1993) strongly suggest that 5-FU plays the preponderant role in the cisplatin–5-FU combination.

*© Cancer Research Campaign 1999* 

The impact of 5-FU catabolism within the target cell in 5-FU resistance had already been pointed out by investigating DPD activity in tumour cells. Experimental data on 19 human cancer cell lines exhibiting spontaneous sensitivity to 5-FU showed that the smaller the DPD activity, the greater the 5-FU sensitivity (Beck et al, 1994). This relationship was further confirmed at the clinical level on 62 HNSCC patients (Etienne et al, 1995). In the present study, tumoural DPD activity was not significantly different between responding and non-responding patients, nor was it significant as a survival indicator. In our previous study (Etienne et al, 1995), the predictive value of DPD was demonstrated after normalization of the tumoural DPD activity by the activity measured in normal tissue. Such normalization (determination of the tumoural/non-tumoural activity ratio) was not feasible in the present work for ethical reasons. Present results highlight the fact that a single determination of DPD activity in the tumoural area does not permit prediction of 5-FU responsiveness.

A recent study by Johnston and colleagues (1997) performed in advanced HNSCC patients suggested that an elevated tumoural TS (immunostaining assay) may be of value in identifying patients who would exhibit resistance to 5-FU-based induction chemotherapy, both in terms of treatment failure and survival. In the present study, tumoural TS activity was unable to predict patient survival or treatment efficacy. Unexpectedly, median TS activity values were higher in complete responders as compared to non-complete responders, even though this difference was not significant (Table 2). Several considerations may explain this observation. Previous data in breast (Pestalozzi et al, 1997) and colorectal cancer (Johnston et al, 1994) have shown that the benefit drawn from 5-FU-based chemotherapy was surprisingly greatest in patients whose tumours had high TS expression. Also, the method chosen by Johnston and colleagues (1997) in HNSCC patients for TS determination differs markedly from ours, since they used an immunohistochemical assay with visual high observer-dependent grading. In addition, this approach does not take into account the active status of the TS protein. For instance, it has been shown that the TS protein can bind to p53 mRNA in vitro (Chu and Allegra, 1996). In our study, we used a biochemical method allowing the TS enzymatic activity itself to be measured. The role of TS for predicting treatment efficacy in HNSCC patients receiving 5-FU-based induction chemotherapy deserves to be further analysed, with special attention being paid to the analytical TS assay.

p53 protein plays a central role in cell cycle control and the apoptotic response to cytotoxic aggressions, particularly radiation (Harris, 1996; Hasegawa et al, 1997). Koch and co-workers (1996) recently showed that mutations of the *p53* gene are associated with an increased risk of loco-regional failure in HNSCC patients treated with radiotherapy. In the present study, we indirectly estimated *p53* mutations by measuring the cellular retention of the p53 protein (immunoluminometric assay) which has been demonstrated to be markedly increased in p53-mutated cells (Raybaud-Diogène et al, 1996). The sequencing of the gene following polymerase chain reaction (PCR) would constitute a better approach for studying p53 in HNSCC (Koch et al, 1996), but this method would be too cumbersome to be applied on large series of patients. Among the 82 patients investigated in the present study, intratumoural levels of p53 protein exhibited tremendous variability, with levels ranging from the sensitivity limit (0.002 ng $ml^{-1}$) up to 24.56 ng $ml^{-1}$ (median 0.19). In line with the data reported by Koch and colleagues (1996), p53 levels were lower in complete responders as compared to non-complete responders, with a statistics close to significance only in the group of patients receiving radiotherapy (RChem group, Table 2). This result highlights the role of p53 status with regard to the cytotoxic effects of radiation therapy (Hasegawa et al, 1997). More importantly, the prognostic value of p53 was tested on specific survival, the analysis being stratified on treatment group (Chem vs Rchem). Analysed as a continuous variable, p53 protein level carries a significant prognostic value, the lower the p53, the longer the survival (Table 3). This finding is in full agreement with previous studies recently performed in HNSCC patients (Raybaud-Diogène et al, 1996). An optimal cut-off value of p53 was defined at 0.80 ng $ml^{-1}$: for the 25 patients (i.e. 30.5%) exhibiting a p53 over 0.80 ng $ml^{-1}$, the risk of death was 2.37-fold that of patients with p53 below 0.80 ng $ml^{-1}$ (Table 3, Figure 2).

Apart from the well-established prognostic value of T staging, the main indicators which showed a high prognostic value in HNSCC over the last 5 years are p53 (Raybaud-Diogène et al, 1996) and EGFR (Dassonville et al, 1993; Störkel et al, 1993; Maurizi et al, 1996). So far, these two parameters have been explored separately and have never been analysed together in a multiparametric approach for predicting outcome in HNSCC patients. This was the main objective of the present study. We presently confirmed the prognostic value of EGFR status (i.e. below or over 220 fmol $mg^{-1}$), specific survival increasing when EGFR was below the threshold (Figure 3). Importantly, when T staging, p53 status and EGFR status were entered in a multivariate Cox analysis stratified on treatment group, the only independent predictors of specific survival were T staging and EGFR status (Table 4A). Also, EGFR status remained a strong prognostic parameter when global response was taken into account in the multivariate Cox analysis (Table 4B).

The disappearance of the prognostic value of p53 in the presence of EGFR may be explained by the demonstrated positive relationship between p53 levels and EGFR (Figure 1). This observation corroborates a previous work in human oesophageal squamous carcinomas showing an EGFR overexpression in tumours with *p53* mutations (Rude Voldborg et al, 1997). This observation is also in agreement with experimental data by Ludes-Meyers et al (1996) showing that tumour-derived *p53* mutants can transactivate the EGFR promoter. In conclusion, the present multifactorial approach, including both 5-FU sensitivity markers and disease aggressiveness indicators to predict treatment outcome in HNSCC patients receiving 5-FU-based induction therapy, strengthens the high prognostic value of EGFR. This result enhances the potential interest of current EGFR-targeted therapy for improving patient outcome (Mendelsohn et al, 1997), particularly in patients exhibiting high tumoural EGFR levels.

### REFERENCES

Beck A, Etienne MC, Cheradame S, Fischel JL, Formento P, Renée N and Milano G (1994) A role for dihydropyrimidine dehydrogenase and thymidylate synthase in tumor sensitivity to fluorouracil. *Eur J Cancer* **30**: 1517–1522

Chu E and Allegra CJ (1996) The role of thymidylate synthase in cellular regulation. *Adv Enzyme Regul* **36**: 143–163

Dassonville O, Formento JL, Francoval M, Ramaioli A, Santini J, Schneider M, Demard F and Milano G (1993) Expression of epidermal growth factor receptor and survival in upper aerodigestive tract cancer. *J Clin Oncol* **11**: 1873–1878

Dimery IW and Hong WK (1993) Overview of combined modality therapies for head and neck cancer. *J Natl Cancer Inst* **85**: 95–111

Etienne MC, Bernard S, Fischel JL, Formento P, Gioanni J, Santini J, Demard F, Schneider M and Milano G (1991) Dose reduction without loss of efficacy for 5 fluorouracil and cisplatin combined with folinic acid. In vitro study on human head and neck carcinoma cell lines. *Br J Cancer* **63**: 372–377

 © Cancer Research Campaign 1999

Etienne MC, Cheradame S, Fischel JL, Formento P, Dassonville O, Renee N, Schneider M, Thyss A, Demard F and Milano G (1995) Response to fluorouracil therapy in cancer patients: the role of tumoral dihydropyrimidine dehydrogenase activity. *J Clin Oncol* **13**: 1663–1670

Greenblatt MS, Bennett WP, Hollstein M and Harris CC (1994) Mutations in the *p53* tumor suppressor gene: clues to cancer etiology and molecular pathogenesis. *Cancer Res* **54**: 4855–4878

Harari MP (1997) Why has induction chemotherapy for advanced head and neck cancer become a United States community standard of practise? *J Clin Oncol* **15**: 2050–2055

Harris BE, Song R and Soong S (1990) Relationship between dihydropyrimidine dehydrogenase activity and plasma 5-fluorouracil levels with evidence for circadian variation of enzyme activity and plasma drug levels in cancer patients receiving 5-fluorouracil by protracted continuous infusion. *Cancer Res* **50**: 197–201

Harris CC (1996) Structure and function of the *p53* tumor suppressor gene: clues for rational cancer therapeutic strategies. *J Natl Cancer Inst* **88**: 1442–1455

Hasegawa M, Mitsuhashi N, Yamakawa M, Furuta M, Maebayashi K, Imai R, Hayakawa K and Niibe H (1997) p53 protein expression and radiation-induced apoptosis in human tumors transplanted to nude mice. *Radiat Med* **15**: 171–176

Johnston PG, Fischer ER, Rockette HE, Fischer B, Wolmark N, Drake JC, Chabner BA and Allegra CJ (1994) The role of thymidylate synthase expression in prognosis and outcome of adjuvant chemotherapy in patients with rectal cancer. *J Clin Oncol* **12**: 2640–2647

Johnston PG, Mick R, Recant W, Beham KA, Dolan ME, Ratain MJ, Beckmann E, Weichselbaum RR, Allegra CJ and Vokes EE (1997) Thymidylate synthase expression and response to neoadjuvant chemotherapy in patients with advanced head and neck cancer. *J Natl Cancer Inst* **89**: 308–313

Koch WM, Brennan JA, Zahuvak M, Goodman SN, Westra WH, Schwab D, Yoo GH, Lee DJ, Forastiére AA and Sidransky D (1996) p53 mutation and locoregional treatment failure in head and neck squamous cell carcinoma. *J Natl Cancer Inst* **88**: 1580–1586

Lejebvre JL, Chevalier D, Luboinski B, Kirkpatrick A, Colletti L and Sahmoud T (1996) Larynx preservation in pyriform sinus cancer. Preliminary results of a European Organization for Research and Treatment of Cancer phase III trial. *J Natl Cancer Inst* **88**: 890–899

Ludes-Meyers JH, Subler MA, Shirakumar CV, Munoz RN, Jiang P, Bigger JE, Brown DR, Deb SP and Deb S (1996) Transcriptional activation of the human epidermal growth factor receptor promoter by human p53. *Mol Cell Biol* **16**: 6009–6019

Mauzizi M, Almadori G, Ferrandina G, Distefano Ml, Romanini ME, Cadoni G, Benedetti-Panici P, Saludetti B, Scambia G and Mancuso S (1996) Prognostic significance of epidermal growth factor receptor in laryngeal squamous cell carcinoma. *Br J Cancer* **74**: 1253–1257

Mendelsohn J (1997) Epidermal growth factor receptor inhibition by a monoclonal antibody as anticancer therapy. *Clin Cancer Res* **3**: 2703–2707

Pestalozzi BC, Peterson HF, Gelber RD, Goldhirsch A, Gusterson BA, Trihia H, Lindtner J, Cortes-Funes H, Simmoncini E, Byzne MS, Gorlouh R, Rudenstam CM, Castighione-Gertsh M, Allegra CJ and Johnston PG (1997) Prognostic importance of thymidylate synthase expression in early breast cancer. *J Clin Oncol* **15**: 1923–1931

Raybaud-Diogène H, Tetu B, Morenay R, Fortin A and Monteil RA (1996) p53 overexpression in head and neck squamous cell carcinoma; review of the literature. *Oral Oncol, Eur J Cancer* **32B**: 143–149

Rude Voldborg B, Damstrup L, Spang-Thomsen M, Skovgaard and Poulsen H (1997) Epidermal growth factor receptor (EGFR) and EGFR mutations, function and possible role in clinical trials. *Ann Oncol* **8**: 1197–1206

Santini J, Milano G, Thyss A, Renée N, Viens P, Ayela P, Schneider M and Demard F (1989) 5-FU therapeutic monitoring with dose adjustment leads to an improved therapeutic index in head and neck cancer. *Br J Cancer* **59**: 287–290

Santini J, Formento JL, Framcoual M, Milano G, Schneider M, Dastonville O and Demard F (1991) Characterization, quantification and potential clinical value of the epidermal growth factor receptor in head and neck squamous cell carcinomas. *Head Neck* **13**: 132–139

Spaulding MB, Fischer SG and Wolf GT (1994) Tumor response, toxicity, and survival after neoadjuvant organ-preserving chemotherapy for advanced laryngeal carcinoma. *J Clin Oncol* **12**: 1592–1599

Spears CP and Gustavsson BG (1988) Methods for thymidylate synthase pharmacodynamics: serial biopsy, free and total TS, FdUMP and dUMP, and $H_4$ Pteglu and $CH_2$-$H_4$ Pteglu assays. In *The Expanding Role of Folates and Fluoropyrimidines in Cancer Chemotherapy*, Rustum Y and McGuire JJ (eds), pp. 97–104. Plenum Press, New York

Störkel S, Reichert T, Reiffen KA and Wagner W (1993) EGFR and PCNA expression in oral squamous cell carcinomas – a valuable tool in estimating the patient’s prognosis. *Eur J Cancer* **29B**: 273–277

Vokes EE, Weichselbaum RR, Lippman SM and Hong WK (1993) Head and neck cancer. *N Engl J Med* **328**: 184–194

*© Cancer Research Campaign 1999*
